# Model of electrical activity in cardiac tissue under electromagnetic induction

**DOI:** 10.1038/s41598-016-0031-2

**Published:** 2016-12-23

**Authors:** Fuqiang Wu, Chunni Wang, Ying Xu, Jun Ma

**Affiliations:** Department of Physics, Lanzhou University of Technology, Lanzhou, 730050 China

## Abstract

Complex electrical activities in cardiac tissue can set up time-varying electromagnetic field. Magnetic flux is introduced into the Fitzhugh-Nagumo model to describe the effect of electromagnetic induction, and then memristor is used to realize the feedback of magnetic flux on the membrane potential in cardiac tissue. It is found that a spiral wave can be triggered and developed by setting specific initials in the media, that is to say, the media still support the survival of standing spiral waves under electromagnetic induction. Furthermore, electromagnetic radiation is considered on this model as external stimuli, it is found that spiral waves encounter breakup and turbulent electrical activities are observed, and it can give guidance to understand the occurrence of sudden heart disorder subjected to heavily electromagnetic radiation.

## Introduction

Accompanied by rhythmical relaxation of heart, complex electrophysiological activities^1–9^ can be detected in cardiac tissue. Spatial pattern^10–14^ can be reproduced and observed by collecting the sampled membrane potentials in different areas of cardiac tissue, and many theoretical models^15–21^ have been proposed to investigate the emergence, phase transition and selection of these spatial patterns. It is believed that local heart ischemia can generate “defects” which can block the propagation of target wave emitted from the sinoatrial node^22^; as a result, self-sustained spiral wave can be induced to block the normal wave propagation. Furthermore, the instability and breakup of spiral wave^23–25^ in cardiac tissue and can cause possible heart disease. Therefore, many schemes^26–31^ have been proposed to remove and suppress the spiral wave in cardiac tissue and chemical media. As mentioned in ref. 29, the external forcing can change the excitability of the media thus the spiral wave can be suppressed in a possible way. On the other hand, external field has also been confirmed to be effective in suppressing spiral wave and turbulence, and the possible mechanism can be associated with depolarization effect. The authors in refs 30 and 31 proposed a scheme of phase compression to suppress the spiral wave in excitable and oscillatory media, particularly, the control mechanism is confirmed as a class of intermittent feedback scheme. Indeed, it is more difficult to suppress and control the pinned spiral wave than the meandering spiral wave because these pinned spiral waves are often attracted to a local area such as heterogeneity. As a result, Zhang and Chen *et al.* ^32–35^ proposed some effective schemes to suppress the pinned spiral wave, and the tip dynamics of spiral wave^36^ has also been discussed.

Indeed, these cardiac tissue models used to emphasize the effect of ion currents across trans-membrane and the membrane potential is calculated, while the effect of electromagnetic induction is left out. As mentioned in refs 37 and 38, complex electrophysiological activities can induce time-varying electromagnetic field and thus the effect of electromagnetic induction on the membrane potential should be considered. Therefore, magnetic flux is proposed to model the effect of electromagnetic induction on cell, and multiple modes of electrical activities can be detected to be consistent with the biological results. In this paper, magnetic flux across the membrane is used to describe the electromagnetic induction and spatial distribution for magnetic flux will be considered, the collective behaviors for myocardial cells will be detected and analyzed by using pattern dynamics.

## Model descriptions

The two-dimensional cardiac tissue with trans-membrane current described by FitzHugh-Nagumo type is often used to detect excitable dynamics of media, it reads^27,39^,

1 $$\left\{\begin{array}{l}\frac{\partial u}{\partial t}=-ku(u-a)(u-1.0)-uv+{I}_{st}+{D}_{u}{\nabla }^{2}u\\ \frac{\partial v}{\partial t}=(\varepsilon +\frac{v{\mu }_{1}}{u+{\mu }_{2}})[-v-ku(u-a-1.0)]\end{array}\right.$$

where *u* is the trans-membrane potential, *v* represents the slow variable for current, *I*_*st*_ is the external forcing current, *D*_*u*_ measures the coefficient of diffusion. The nonlinear term −*ku*(*u* – *a*)(*u* – 1.0) − *uv* denotes the total trans-membrane ionic currents per unit area. *k, ε, μ*_1_, *μ*_2_ are fixed parameters to set this model so that main properties of excitable media can be reproduced. For example, it often sets *a* = 0.15, *μ*_1_ = 0.2, *μ*_2_ = 0.3, *k* = 8.0, *ε* = 0.002. According the law of electromagnetic induction, time-varying electromagnetic field can be set up during the fluctuation of electrical activities in cardiac tissue. Herein, the magnetic flux can used to measure the changes of electromagnetic field if possible. As a result, the changes of magnetic flux can adjust the spatial distribution of membrane potentials of cells, here; a memristor^40^ is used to realize the coupling and modulation on membrane potential from magnetic flux so that the consistency of physical dimension (or unit) can be kept. Memristor is a new electrical device composed of complex nonlinearity, and it is often used for nonlinear circuits^41–43^ with memory effect because the memductance is dependent on the external forcing current, the nonlinear memductance function^44,45^ for memristor is described by

2 $$\rho (\varphi )=\frac{dq(\varphi )}{d\varphi }=\alpha +3\beta {\varphi }^{2}$$

where *q*(*φ*) is the memristor constitutive relation, the parameters *α, β* are often selected by appropriate values such as *α* = 0.2, *β* = 0.3, so that the memristor-coupled nonlinear circuit can generate chaotic state. When the effect of electromagnetic induction is considered, the dynamical equations for the three-variable Fitzhugh-Nagumo model are described by

3 $$\left\{\begin{array}{l}\frac{\partial u}{\partial t} =  -ku(u-a)(u-1.0)-uv+{k}_{0}\rho (\varphi )u+{D}_{u}{\nabla }^{2}u\\ \frac{\partial v}{\partial t}  =  (\varepsilon +\frac{v{\mu }_{1}}{u+{\mu }_{2}})[-v-ku(u-a-1.0)]\\ \frac{\partial \varphi }{\partial t}  =  {k}_{1}u-{k}_{2}\varphi \end{array}\right.$$

The third variable *φ* describes the magnetic flux, the *ρ*(*φ*) is a flux-controlled memristor and calculates the effect of magnet-controlled memristor. The parameter *k*_0_, *k*_1_ *k*_2_ are gains used to calculate the effect of electromagnetic induction on cells. The term *k*_0_ *ρ*(*φ*)*u* describes the relation of modulation on membrane potential, and it is dependent on the variation in magnetic flux by generating additive faradic current. In the following section, the spatial pattern selection is calculated by discerning the distribution for membrane potentials in numerical way, and this media is considered in two-dimensional array space composed of 200 × 200 nodes.

## Numerical Results and Discussions

**Figure 1:**
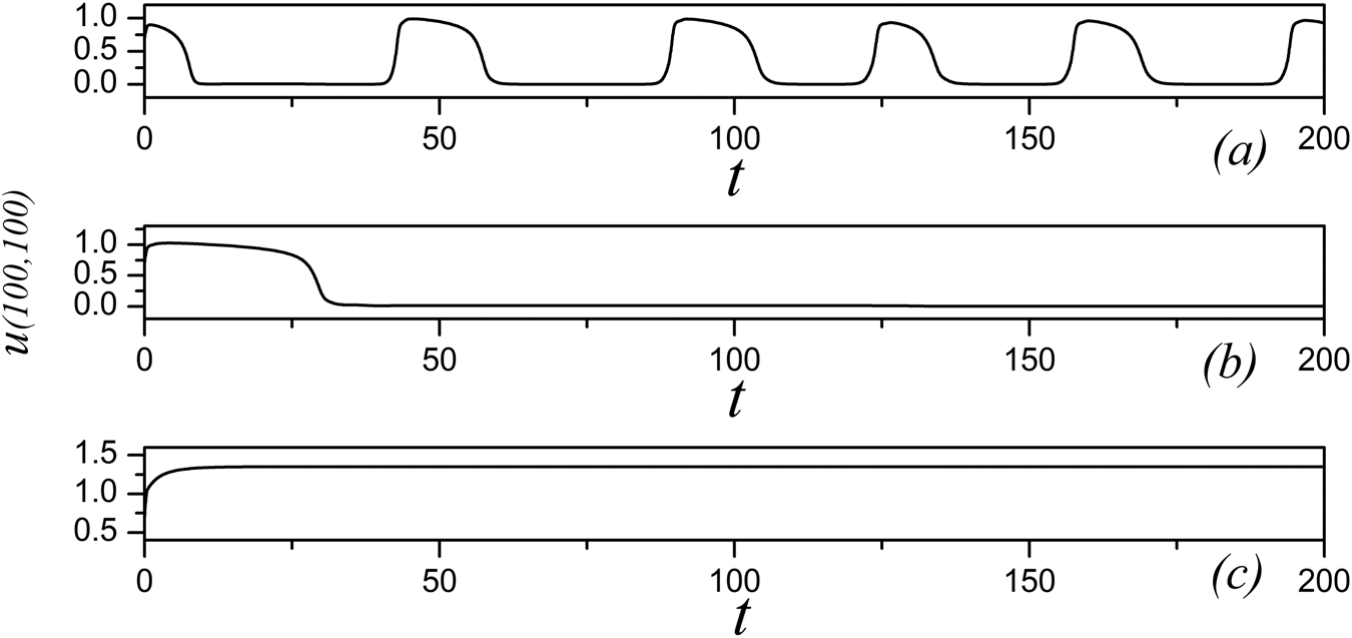
The time series of membrane potential are detected from the node (100,100) under different gains *k*_*0*_ at *k*_1_ = 0.5, *k*_2_ = 1, *α* = 1, *β* = 2, for (**a**) *k*_*0*_ = 0.1; (**b**) *k*_*0*_ = 0.4; (**c**) *k*_*0*_ = 0.9.

In the numerical studies, the Euler forward algorithm is used with time step *h* = 0.03 and no-flux boundary condition being used. The membrane potential series are calculated by selecting different parameters and gains for fixed *k*_0_, *k*_2_, *α, β*, thus different electrical modes can be triggered. In case of pattern formation and selection, the size of the media is set as 350 × 350, the space unit is calculated as 350/200, the diffusive coefficient *D*_*u*_ is set at 1 for simplicity. The initial values are selected as *u*(92:97,1:115) = 1.0, *v*(92:97,1:115) = 0.0, *φ*(92:97,1:115) = 0.0; *u*(98:103,1:115) = 0.7, *v*(98:103,1:115) = 0.6, *φ*(98:103,1:115) = 0.1; *u*(104:109,1:115) = 0.0, *v*(104:109,1:115) = 0.8, *φ*(104:109,1:115) = 0.2; the other nodes are set as (*u, v*, *φ*) = (0, 0, 0), the external forcing current *I*_*st*_ = 0.0. To be consistent with the previous works, the parameters are selected as, *k*_1_ = 0.5, *k*_2_ = 1, *α* = 1.0, *β* = 2.0. At first, the sampled membrane potentials for certain node (100, 100) are detected to generate different modes of electrical activities by selecting appropriate gain for *k*_0_, and the results are shown in Fig. 1.

**Figure 2:**
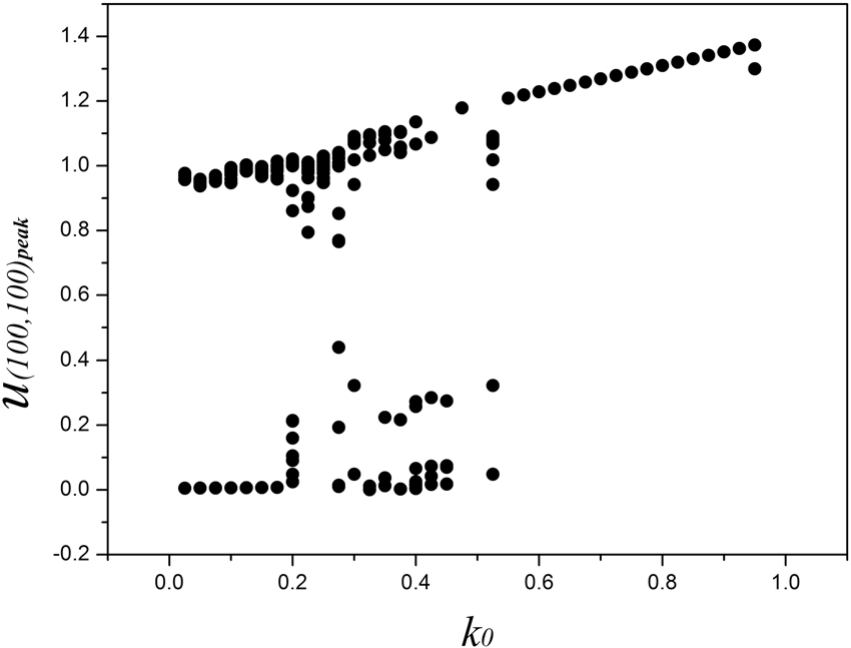
Bifurcation diagram for maximal membrane potential vs. the gain *k*_0_, the parameters are set as *k*_1_ = 0.5, *k*_2_ = 1, *α* = 1, *β* = 2. Within the sampled time series for membrane potential for node (100, 100), the peak value for membrane potential is recorded only when the membrane potential is beyond the values for the pre-adjacent and post-adjacent time.

It is found in Fig. 1 that the spiking and quiescent state can be observed by selecting appropriate gain *k*_0_, and it indicates that the dynamical properties of membrane potential depend on the modulation resulting from the magnetic flux. Furthermore, the bifurcation diagram for the maximal membrane potential on node (100, 100) vs. feedback gain *k*_0_ is calculated in Fig. 2.

**Figure 3:**
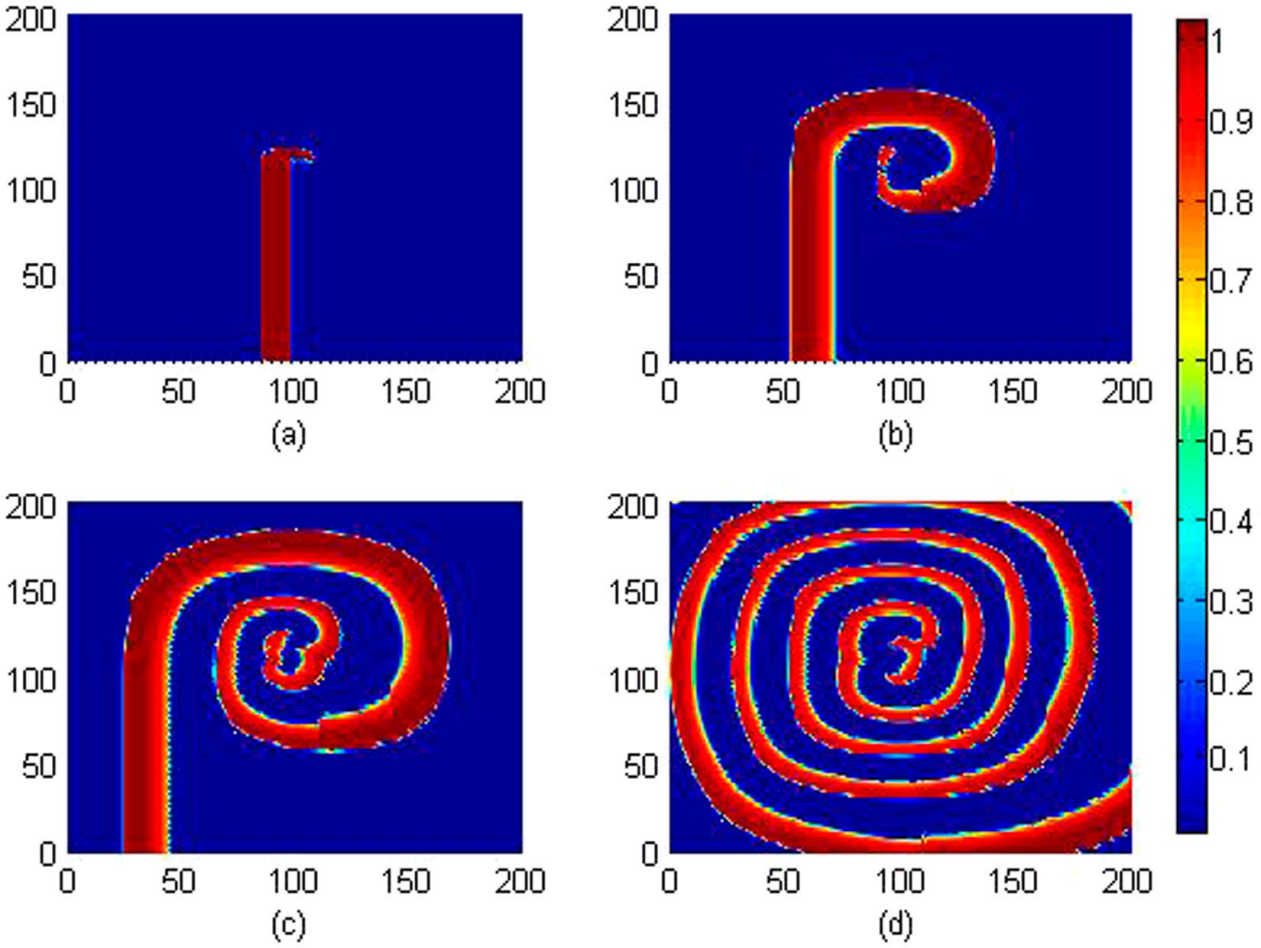
Developed spatial pattern is plotted for the media at *t* = 10, 60, 100, 200 time units under feedback gain *k*_0_ = 0.1, respectively. The snapshots are shown color scale.

The bifurcation diagram in Fig. 2 confirmed that multiple peaks can be found in the sampled time series for membrane potentials and thus multiple modes in in electrical activities of cardiac tissue can be induced under appropriate feedback gain. Furthermore, the feedback gain *k*_0_ is slowly changed, and the wave emergence and pattern selection are calculated to detect the development of collective behaviors, the evolution and development of patterns are plotted in Fig. 3.

**Figure 4:**
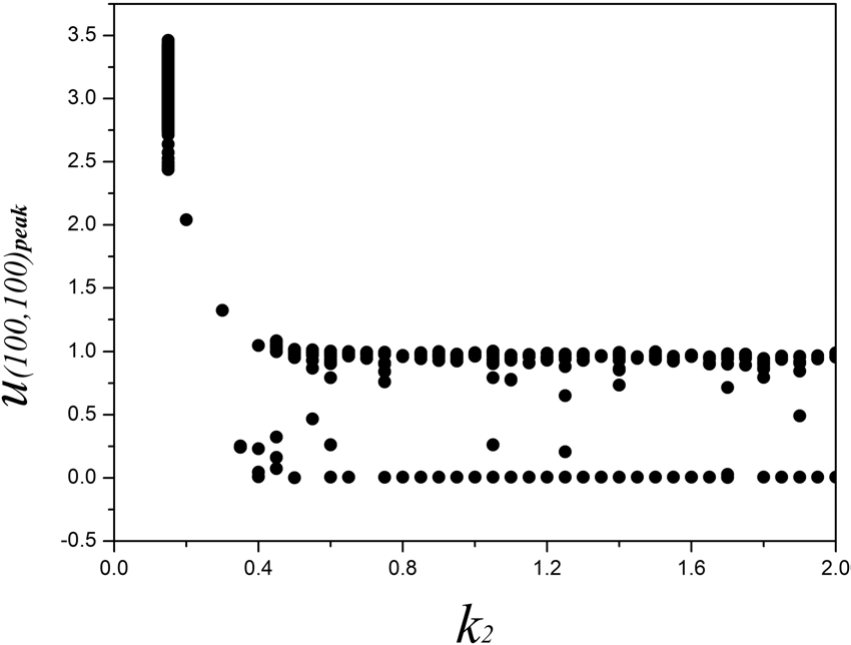
Bifurcation diagram for maximal membrane potential vs. ***k***_**2**_ , **the parameters are set as** ***k***_**0**_ **= 0.1** , ***k***_**1**_ **= 0.5** , ***α*** **= 1** , ***β*** **= 2**.

It is confirmed that stable spiral waves can be induced in the media by setting appropriate initials and fixed parameters, and these results are similar to the case when no electromagnetic flux is considered. Furthermore, the feedback gain is increased, for example, *k*_0_ = 0.4, 0.9, the same initials and parameters setting can’t induce and support the survival of spiral waves. The potential mechanism could be that larger feedback gain *k*_0_ imposes stronger modulation on membrane potentials due to the effect of electromagnetic induction, and the media is magnetized for homogeneous state. It also indicates that magnetic field could be helpful to suppress the spiral wave if possible, and it is important to verify this assumption. We also checked the dependence of parameter *k*_2_ on stabilizing the spatial pattern and dynamical properties for sampled membrane potentials, the parameters are selected as *k*_0_ = 0.1, *k*_1_ = 0.5, *α* = 1, *β* = 2, and the bifurcation analysis is calculated in Fig. 4.

**Figure 5:**
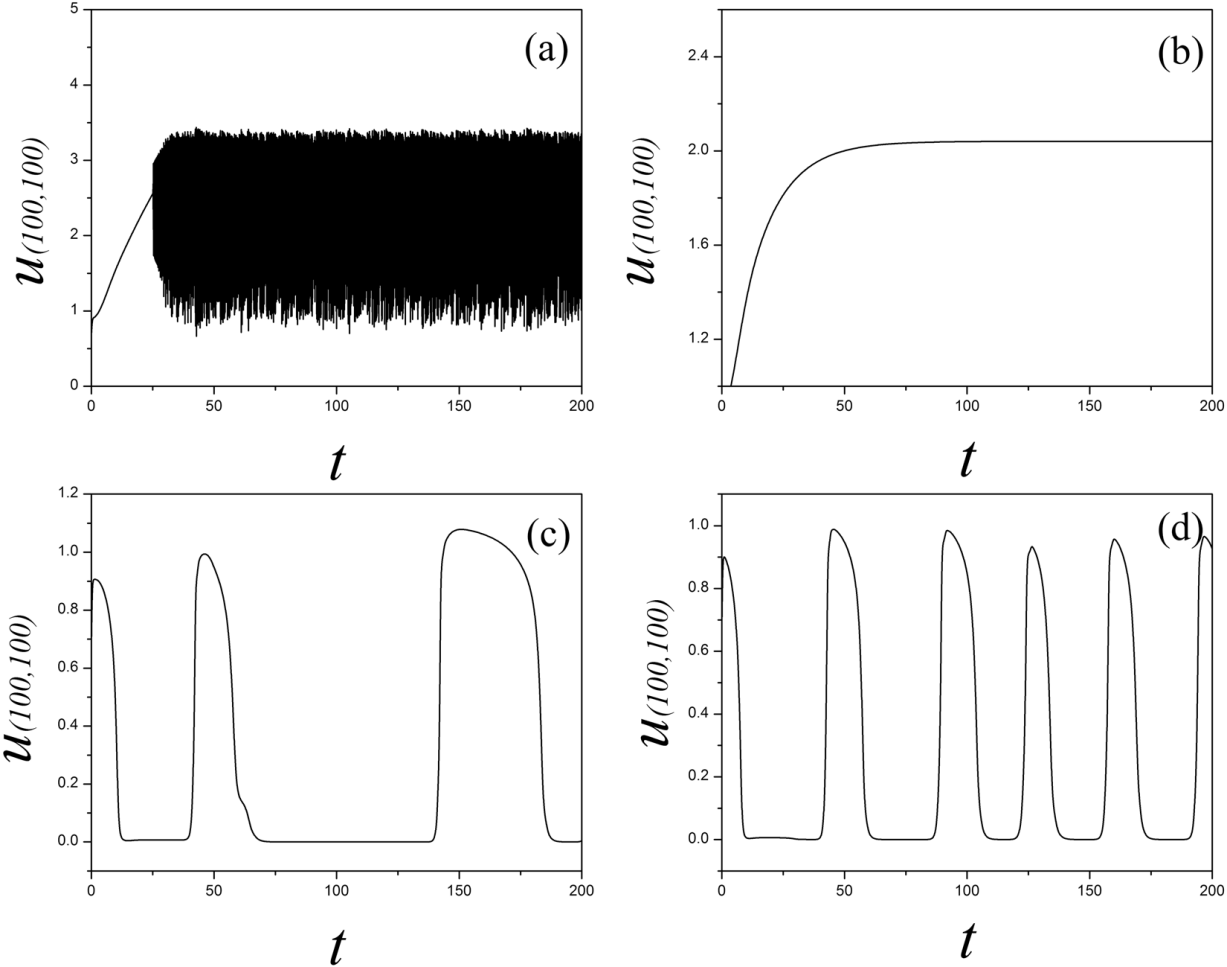
Sampled time series for membrane potential for the node (100, 100) are calculated with different gain *k*_2_ being used, the parameters are set as *k*_0_ = 0.1, *k*_1_ = 0.5, *α* = 1, *β* = 2, for (**a**) *k*_*2*_ = 0.1; (**b**) *k*_*0*_ = 0.2; (**c**) *k*_2_ = 0.5; (**d**) *k*_2_ = 1.0.

It is found that the sampled time series are dependent on the selection of feedback gain *k*_2_. Indeed, larger value for *k*_2_ can stabilize the fluctuation of magnetic flux and even homogeneous state can be developed in distribution of magnetic flux, thus the dynamical properties of the media began to be dependent of the magnetic field. In the case of lower feedback *k*_2_, the sampled time series present chaotic-like behaviors and are verified in Fig. 5.

**Figure 6:**
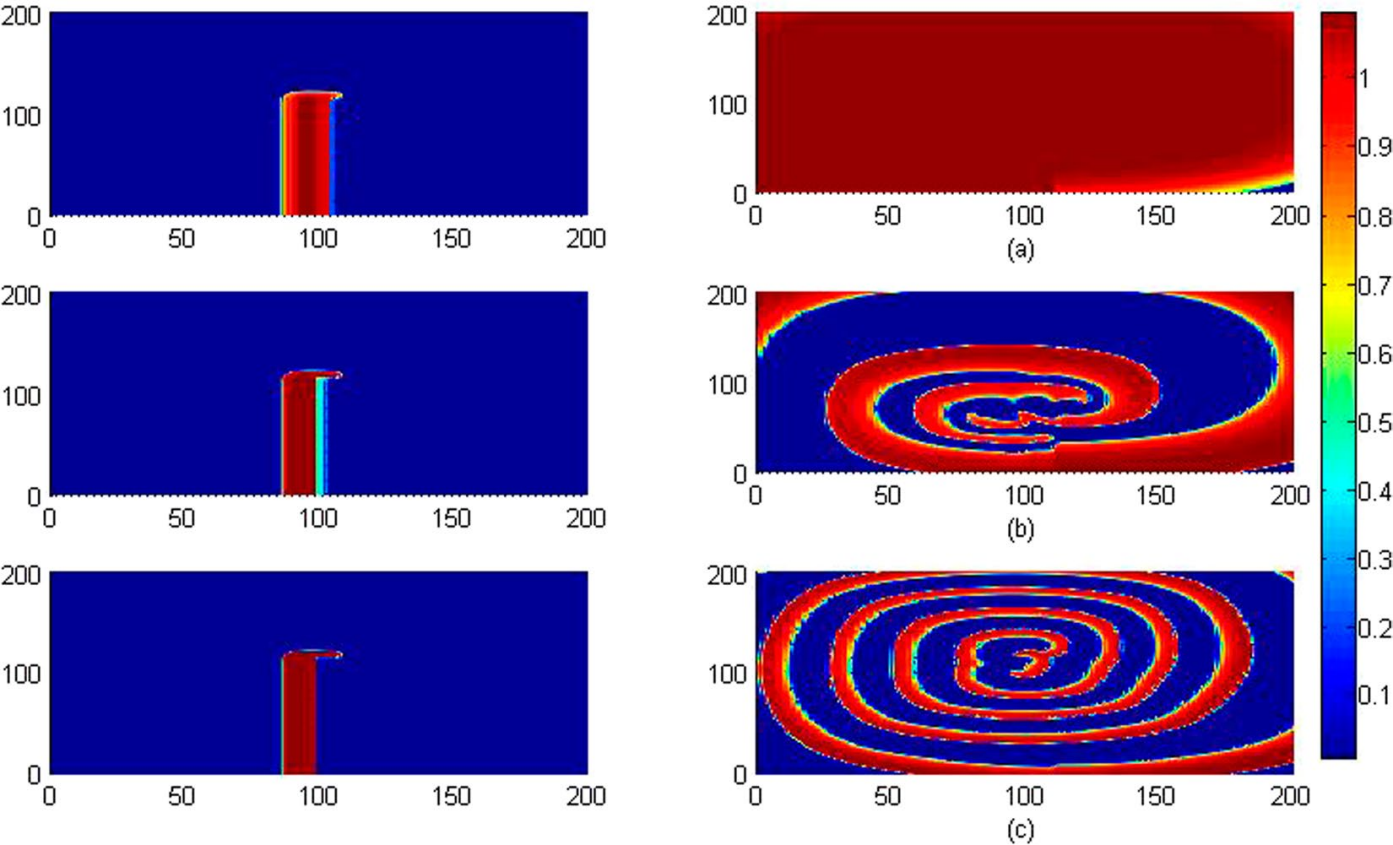
Developed spatial pattern is plotted for the media at *t* = 10, 200 time units under different feedback gain *k*_2_, at *k*_0_ = 0.1, *k*_1_ = 0.5, *α* = 1, *β* = 2, for (**a**) *k*_2_ = 0.2; (**b**) *k*_2_ = 0.5; (**c**) *k*_2_ = 1.0. The snapshots are shown color scale.

By setting different feedback gains *k*_2_, the sampled time series show different modes in electrical activities. The spatial distribution in the media is triggered to generate turbulent, homogeneous states and then regular pattern such as spiral waves can be induced by increasing the feedback gain *k*_2_. Indeed, the sampled time series for the media show distinct periodicity when standing spiral wave is developed because the media can be regulated by the spiral wave completely. For visualized illustration and understanding, the development of spiral wave and patterns are calculated under different feedback gains *k*_2_ setting, and the results are plotted in Fig. 6.

**Figure 7:**
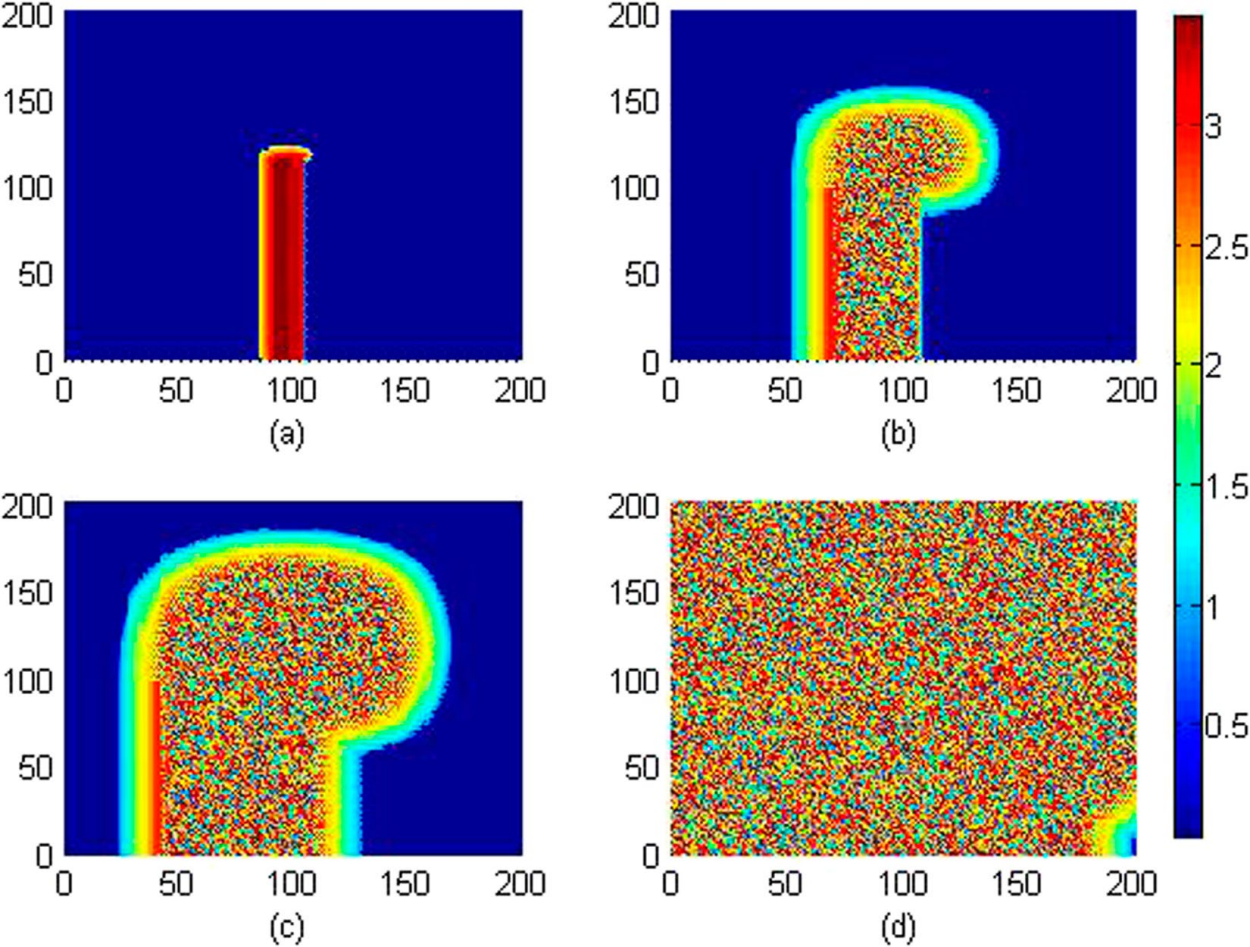
Developed spatial pattern is plotted for the media at *t* = 10, 60, 100, 200 time units under feedback gain *k*_2_ = 0.15, at *k*_0_ = 0.1, *k*_1_ = 0.5, *α* = 1, *β* = 2. The snapshots are shown color scale.

The results in Fig. 6 showed that spiral wave can be induced in the media and the stability of spiral wave is dependent on the selection of feedback gain *k*_2_. In fact, larger value for *k*_2_ can stabilize possible change of the electromagnetic field, changes of magnetic flux, thus the media can be effective to support periodical wave fronts. Furthermore, smaller feedback gain *k*_2_ is applied, and the results for pattern formation are shown in Fig. 7.

**Figure 8:**
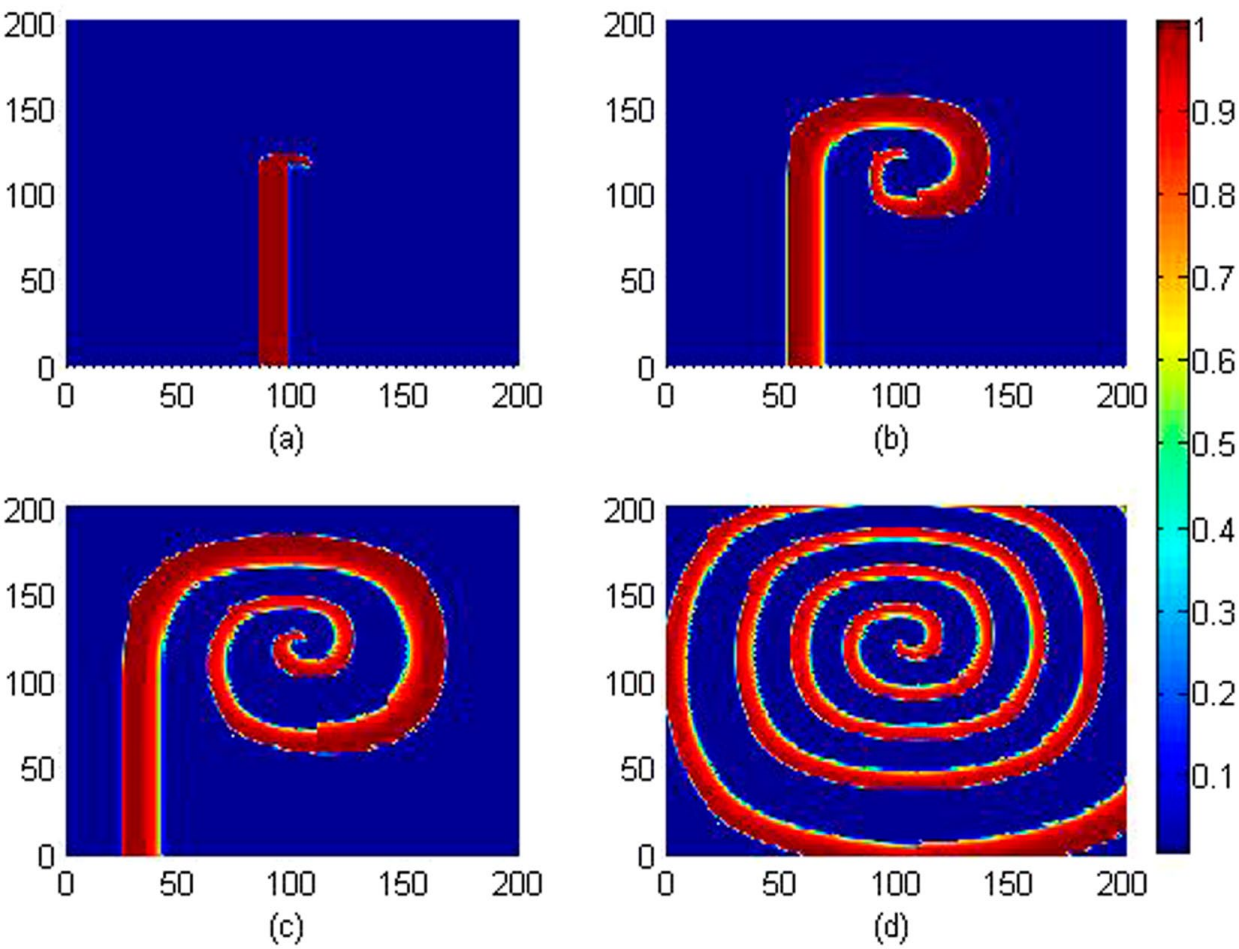
Developed spatial pattern is plotted for the media at *t* = 10, 60, 100, 200 time units under feedback gain *k*_2_ = 3.0, at *k*_0_ = 0.1, *k*_1_ = 0.5, *α* = 1, *β* = 2. The snapshots are shown color scale.

It is found in Fig. 7 that wedged-shape setting for initials ever triggered spiral seeds but failed to develop a perfect spiral wave in the media, particularly, breakup occurs on the spiral seeds and the media becomes turbulent due to diffusive coupling in the media. However, spirals can be formed to develop a standing and perfect spiral wave in the media by further increasing the feedback gain *k*_2_, the results are plotted in Fig. 8.

**Figure 9:**
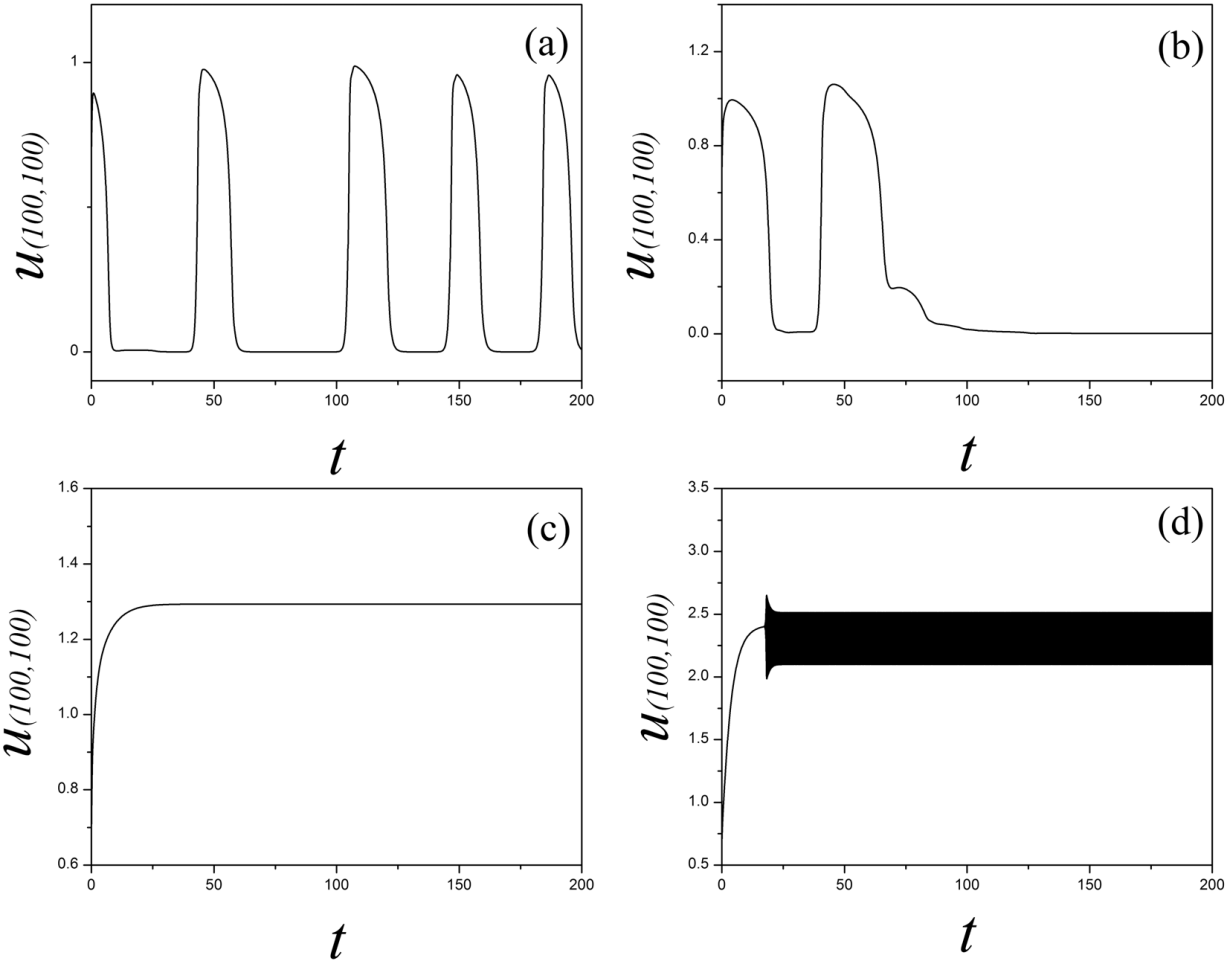
Sampled time series for membrane potential for the node (100,100) with different gain *k*_1_ at *k*_0_ = 0.1, *k*_2_ = 1.0, *α* = 1, *β* = 2, for (**a**) *k*_1_ = 0.2; (**b**) *k*_1_ = 1.2; (**c**) *k*_1_ = 1.6; (**d**) *k*_1_ = 2.7.

**Figure 10:**
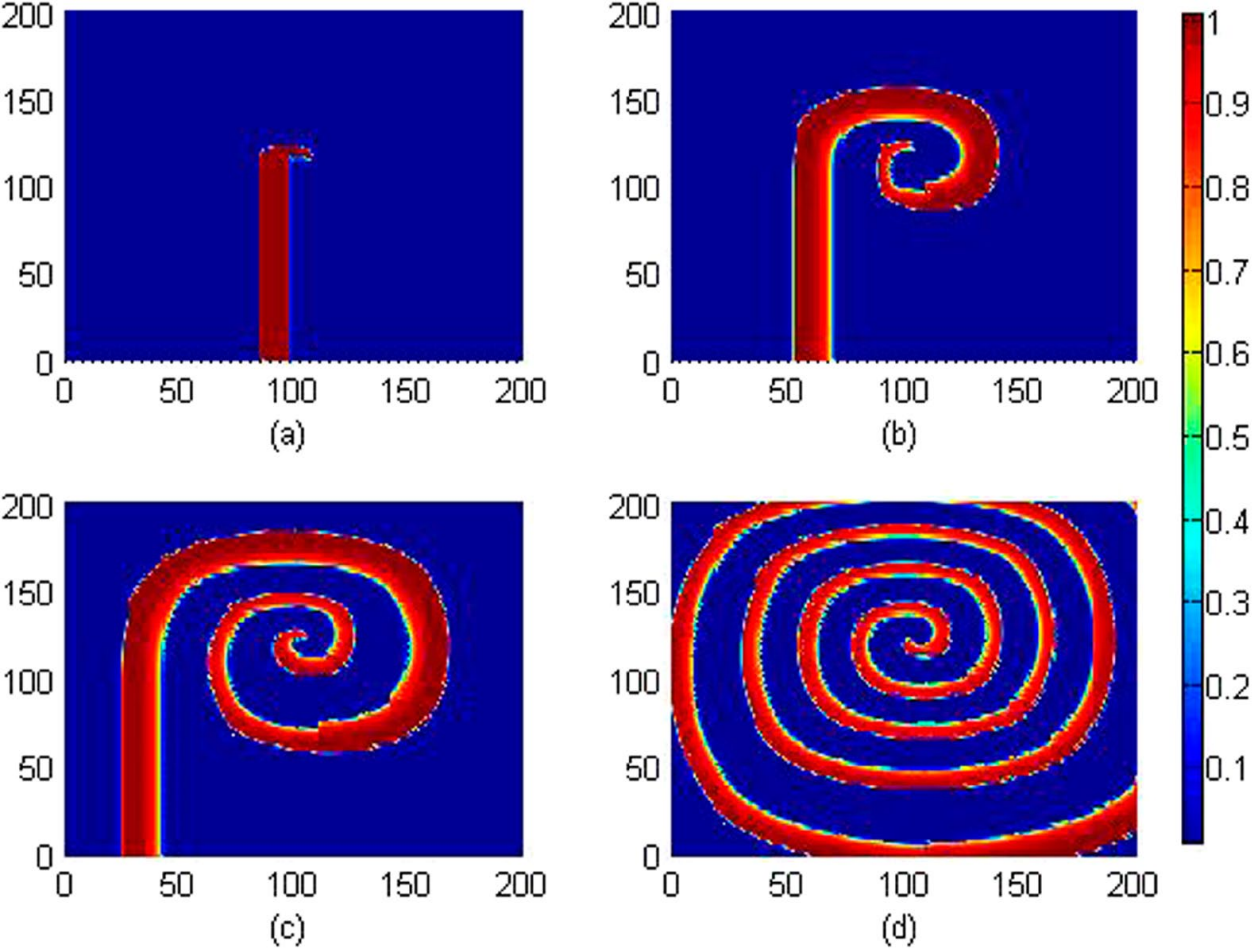
Developed spatial pattern is plotted for the cardiac tissue at *t* = 10, 60, 100, 200 time units under feedback gain *k*_1_ = 0.2, at *k*_0_ = 0.1, *k*_2_ = 1.0, *α* = 1, *β* = 2. The snapshots are shown color scale.

It is found in Fig. 8 that the media with larger feedback gain *k*_2_ is helpful to support the propagation of spiral waves, the potential mechanism could be that the distribution for magnetic flux can be regulated to approach regular distribution under diffusive superposition. Furthermore, we also investigated the case when the media is adjusted by the parameter *k*_1_, which describes the sensitivity of electromagnetic induction in the media, and a larger *k*_1_ indicates that the media is much sensitive to electromagnetic induction and can contribute to the changes of membrane potential of cells greatly. The sampled time series for membrane potentials are calculated in Fig. 9 and pattern development is shown in Fig. 10.

As shown in Figs 9 and 10, smaller value is set for the feedback gain *k*_1_ that the media is excitable for wave propagation and survival, the sampled time series also show distinct periodicity as well when the media is regulated by continuous waves. On the other hand, setting larger value for the feedback gain *k*_1_ can enhance the effect of electromagnetic induction and the membrane potential changes in rapid rhythm which can break the regular spatial distribution, as a result, spiral wave is suppressed. Therefore, setting appropriate feedback gains *k*_1_, *k*_2_ is effective to support pattern formation, and the electromagnetic induction can also destroy spatial distribution and the modes in electrical activities in the media.

To confirm our prediction for the effect of electromagnetic induction, external forcing with electromagnetic field is imposed on local area of the media, and the development of collective behaviors in electrical activities is investigated by detecting the sampled time series and pattern selection. When external electromagnetic field is imposed, the distribution for magnetic flux will be changed, therefore, the effect of electromagnetic field is described by imposing nonlinear function for magnetic flux on the third formula. For simplicity, the parameters are set as *k*_0_ = 0.1, *k*_1_ = 0.5, *k*_*2*_ = 1.6, *α* = 1, *β* = 2, and the external electromagnetic radiation on the media is described by

4 $$\frac{\partial \phi }{\partial t}={k}_{1}u-{k}_{2}\phi +F(x,y,t)$$

where the nonlinear function *F*(*x, y, t*) represents the external electromagnetic radiation, *x, y* denotes the position in the media. For simplicity, it sets *F*(*x, y, t*) = *Aexp*(−*mr*), *r* ^2^ = (*x* − *x*_0_)^2^ + (*y* − *y*_0_)^2^, *A* is the amplitude for electromagnetic radiation and *m* is the gradient factor decreasing the electromagnetic field. (*x*_0_, *y*_0_) is the center of the area exposed to external electromagnetic field.

**Figure 11:**
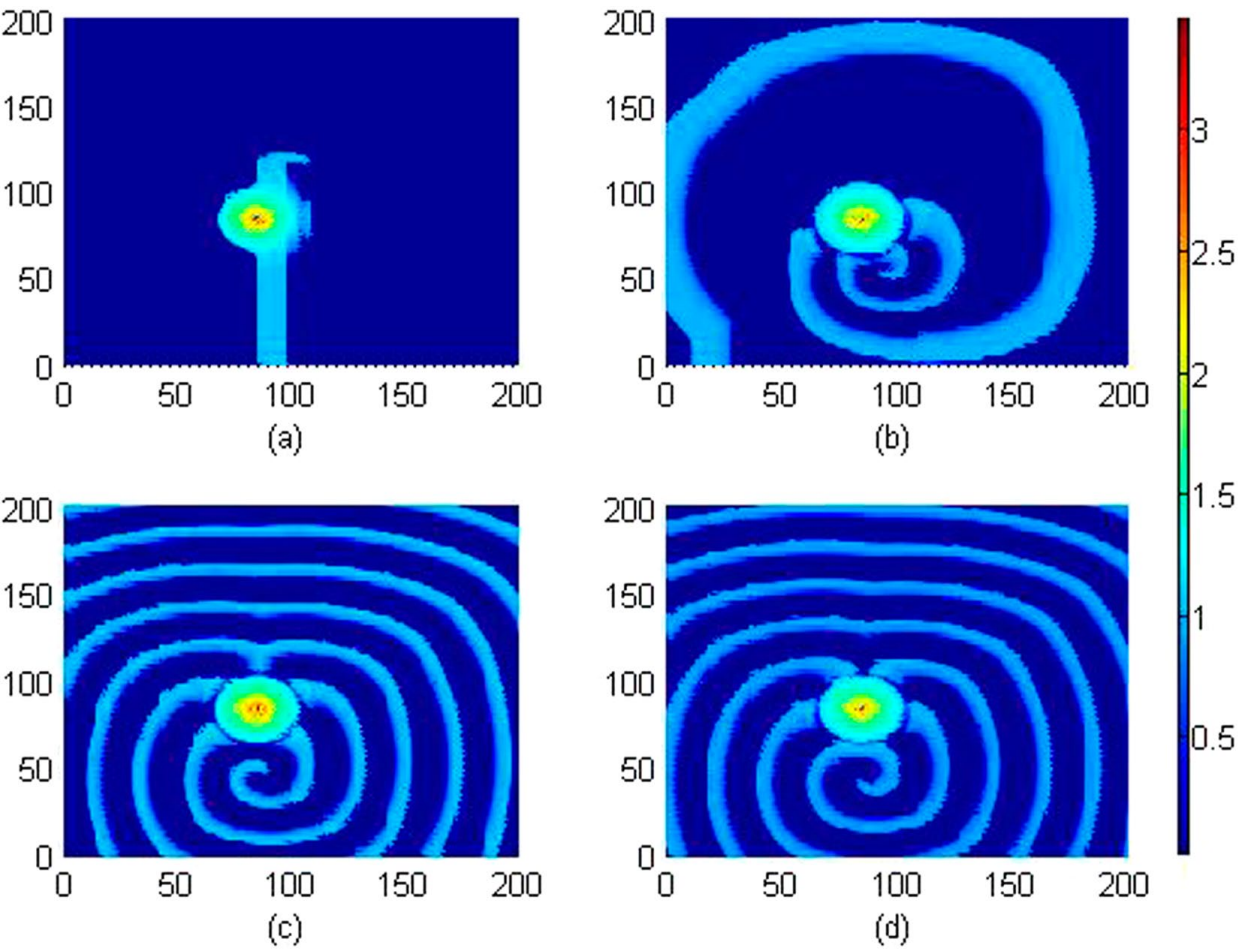
Developed spatial pattern is plotted for the media by setting wedge-shaped initial values, and the center of area exposed to electromagnetic field is selected as (*x*_0_, *y*_0_) = (84,84), at *A* = 12, *m* = 0.06, *k*_0_ = 0.1, ***k***_1_ = 0.5, *k*_2_ = 1.6, *α* = 1, *β* = 2. For *t* = 10 (**a**), 120 (**b**), 600 (**c**), 800 (**d**) time units. The snapshots are plotted in color scale.

**Figure 12:**
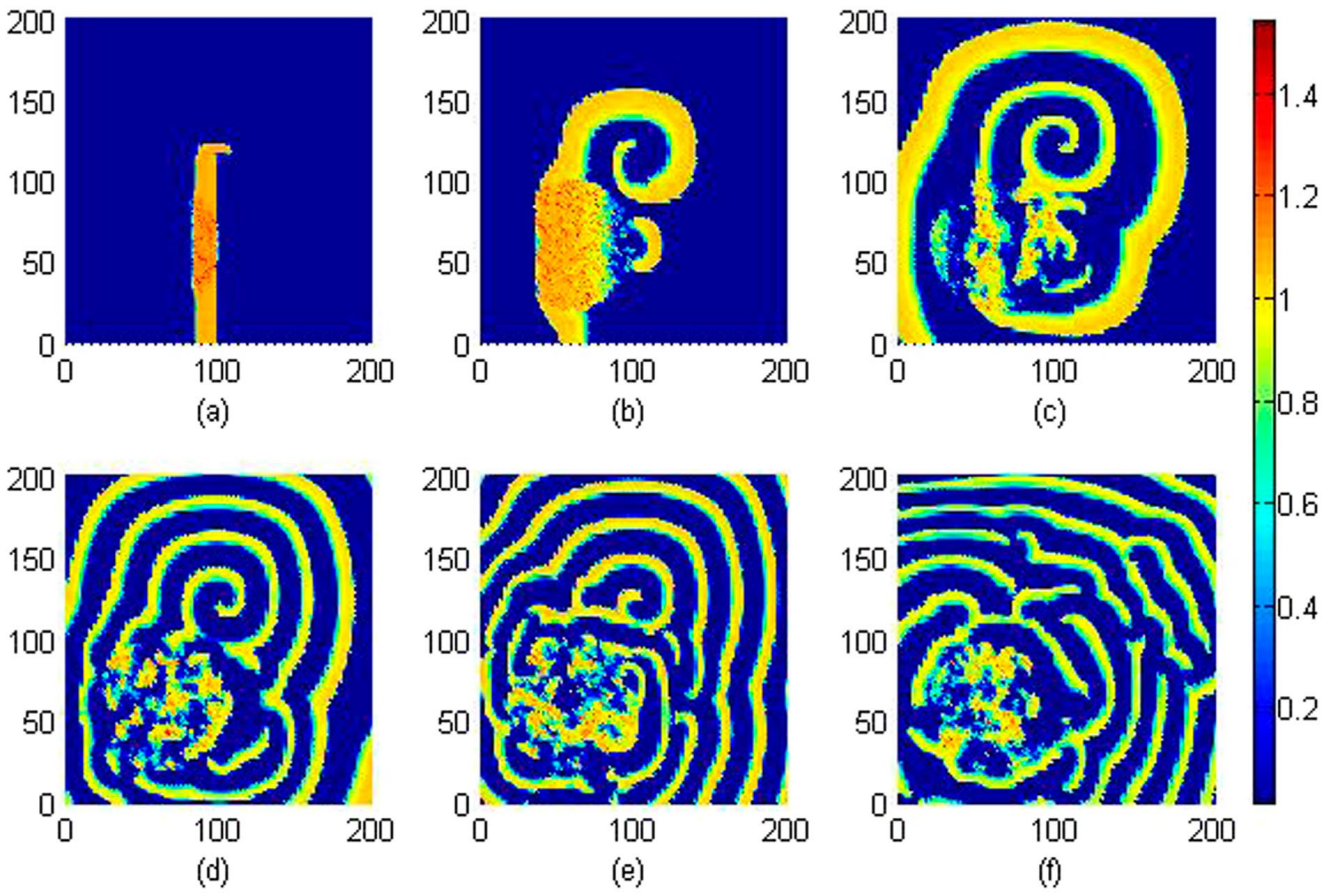
Developed spatial pattern is plotted for the media by setting wedge-shaped initial values, and the center of area exposed to electromagnetic field is selected as (*x*_0_, *y*_**0**_) = (60,60), *r* ≤ 40, noise intensity is set as *D* = 9, and parameters are selected as *k*_0_ = 0.1, *k*_1_ = 0.5, *k*_2_ = 1.6, *α* = 1, *β* = 2. For *t* = 10 (**a**), 60 (**b**), 120 (**c**), 200 (**d**), 400 (**e**), 800 (**f**) time units.

It is confirmed in Fig. 11 that local electromagnetic radiation can block the wave propagation and the developed spiral wave is driven away from the center of the media. Extensive numerical studies were also carried out by setting different beginning area (*x*_0_, *y*_0_) exposed to external electromagnetic radiation, more electromagnetic sources are used to drive the media, and similar results are approached that developed regular distribution can be destroyed and the spiral wave is broken. Furthermore, we considered the case when external electromagnetic radiation is imposed on the media with noise type. For example, the *F*(*x, y, t*) is selected with Gaussian white noise and imposed on the media uniformly, and the results for pattern development are plotted in Fig. 12.

**Figure 13:**
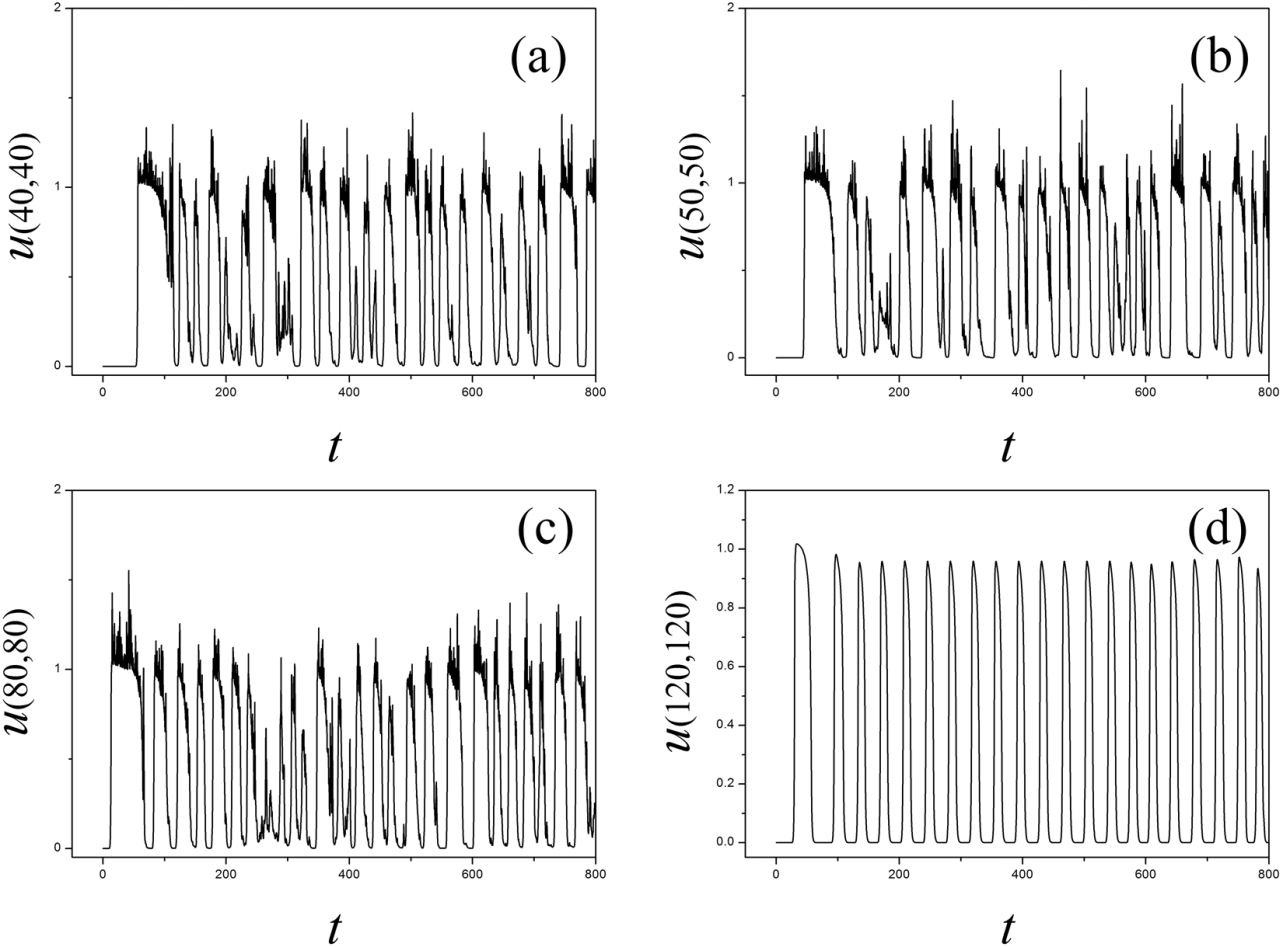
Sampled time series for membrane potentials on different nodes are calculated, and the center of area exposed to electromagnetic field is selected as (*x*_0_, *y*_0_) = (60, 60), noise area *r* ≤ 40, noise intensity is set as *D* = 9, *k*_0_ = 0.1, *k*_1_ = 0.5, *k*_2_ = 1.6, *α* = 1, *β* = 2.

It is confirmed in Fig. 12 that spiral wave can be triggered in local area by setting specific initial values, with increasing the transient period, the spirals are broken when the noise-like electromagnetic radiation is imposed on the media completely. Furthermore, the sampled time series for membrane potentials on different nodes are calculated in Fig. 13.

**Figure 14:**
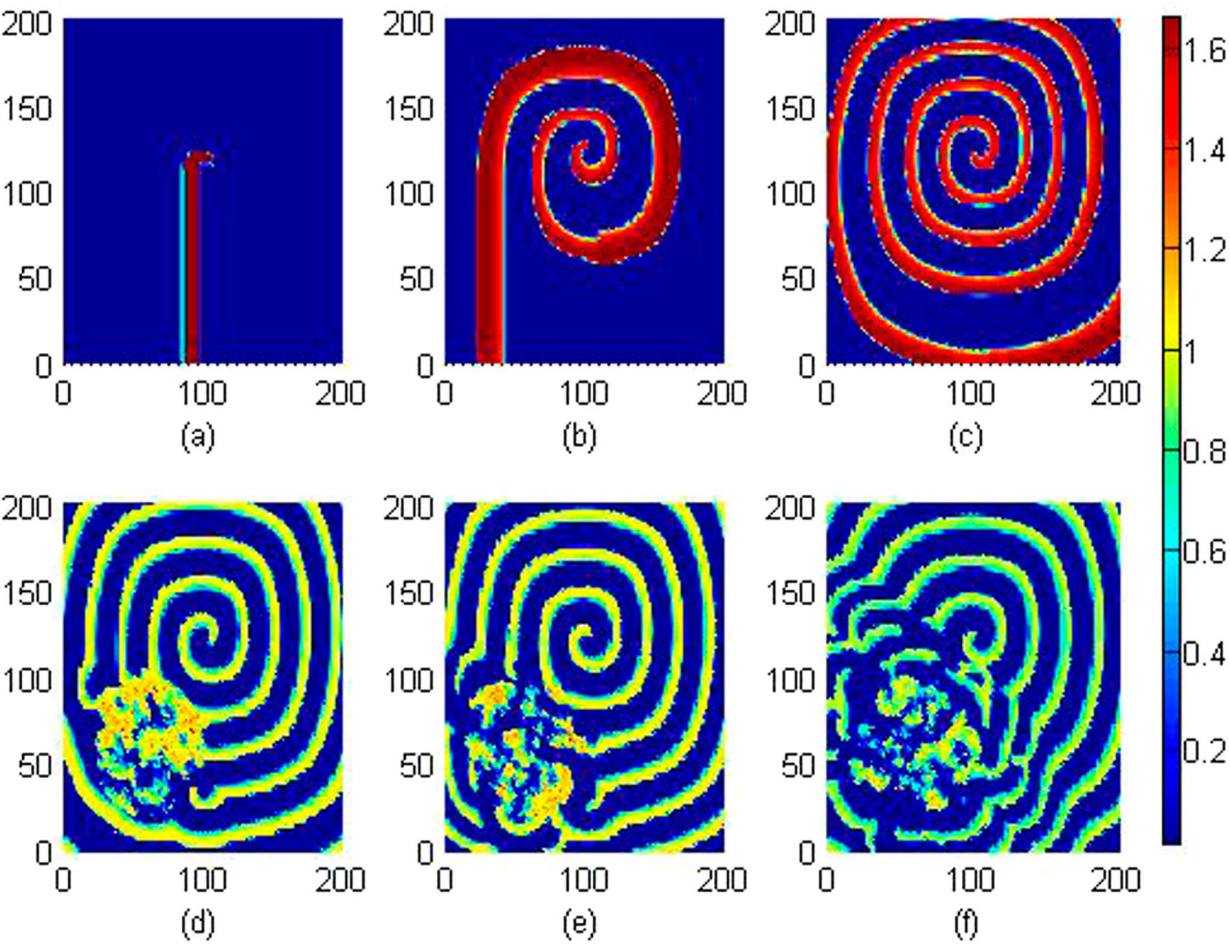
Developed spatial pattern is plotted for the media by setting wedge-shaped initial values within a transient period about 200 time units, and the center of area exposed to electromagnetic field is selected as (*x*_0_, *y*_0_) = (60, 60), noise area *r* ≤ 40, noise intensity is set as *D* = 9, *k*_0_ = 0.1, *k*_1_ = 0.5, *k*_2_ = 1.6, *α* = 1, *β* = 2. For *t* = 10 (**a**), 100 (**b**), 200 (**c**), 300 (**d**), 400 (**e**), 800 (**f**) time units.

**Figure 15:**
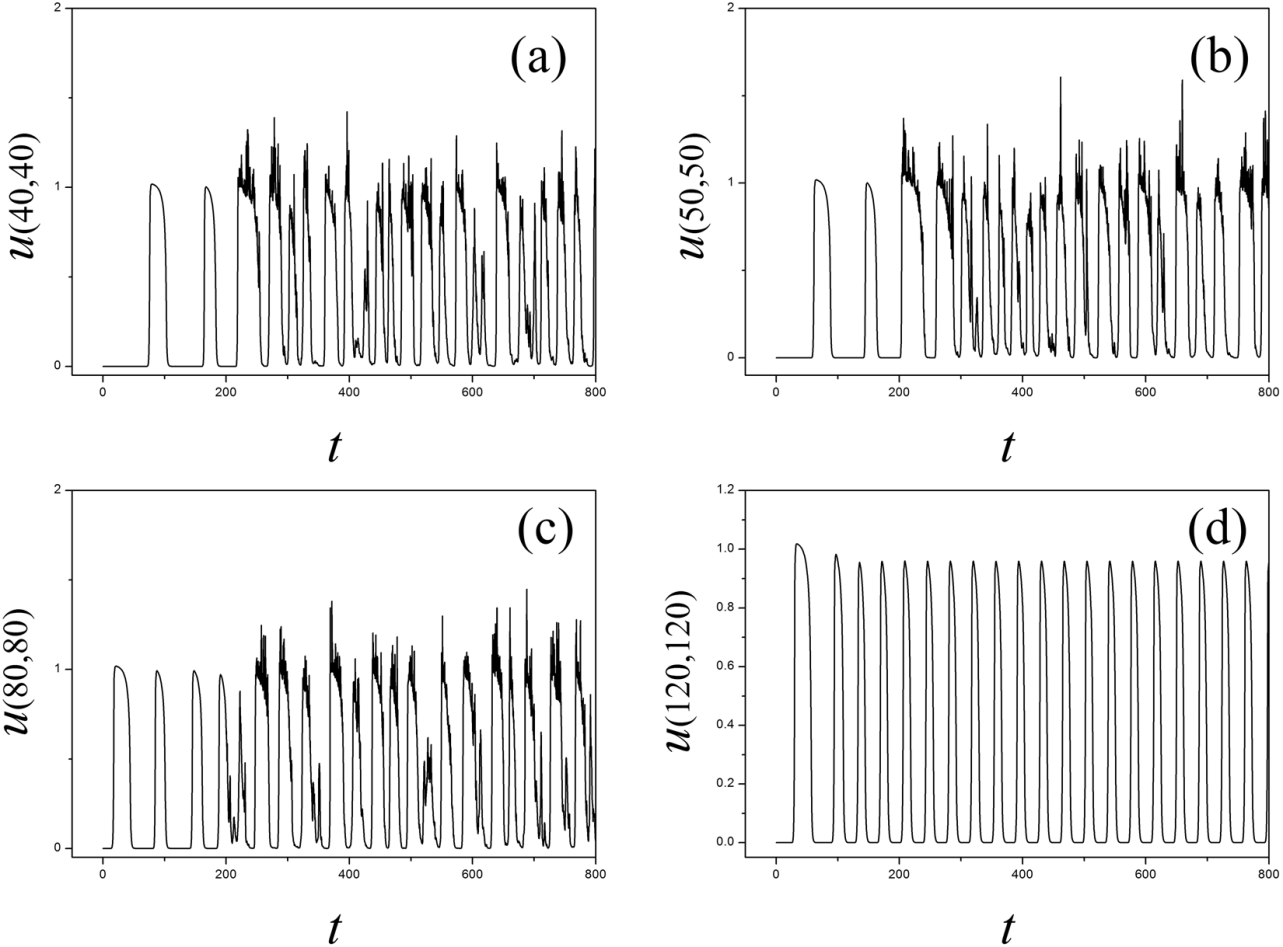
Sampled time series for membrane potentials for different nodes, nd the center of area exposed to electromagnetic field is selected as (*x*_0_, *y*_0_) = (60, 60), noise area *r* ≤ 40, noise intensity is set as *D* = 9, *k*_0_ = 0.1, *k*_1_ = 0.5, *k*_2_ = 1.6, *α* = 1, *β* = 2.

The results in Fig. 13 showed that electrical activities present different modes, for example, spiking state, bursting state and even chaotic state can be observed in the media. That is to say, electromagnetic radiation could be destructive for development and growth of spiral waves. It is also important to investigate the robustness of spiral wave to external electromagnetic radiation. In this case, a stable rotating spiral wave is developed in the media with a transient period about 200 time units and used for initial state to be controlled by Gaussian white noise, the pattern transition is shown in Fig. 14 and the sampled time series for membrane potentials from four different nodes are illustrated in Fig. 15.

It is found In Fig. 14 that spiral waves can be broken and the media began to step into disordered states. In Fig. 15, it is interesting to observe different modes in the electrical activities by imposing noise-like electromagnetic radiation on local area, the nodes belonged to the area driven by noise generate time series for membrane potentials in irregular rhythm because this area becomes turbulent resulting from the instability of spiral waves. Above all, a new cardiac tissue model is set up with the effect of electromagnetic induction being considered. The distribution for magnetic flux in the media plays important role in changing the dynamical properties in electrical activities and also the wave propagation, pattern formation in the media.

Finally, it is important to clarify the importance of memristor and magnetic flux within the presented model. Magnetic flux describes the effect of electromagnetic induction and also the changes of electromagnetic field as well. Memristor can measure the time-varying relation between magnetic flux across the membrane and ion currents, and thus the modulation of magnetic flux on membrane potential can be mapped. The most novelty of this work can be that effect of electromagnetic induction is considered by introducing the physical magnetic flux, memristor is used to map the modulation of electromagnetic induction on membrane potential. As a result, the external electromagnetic radiation on the media can be understood by changing the distribution of electromagnetic field and then the membrane potential.

## Conclusions

Based on the law of electromagnetic induction, the cardiac tissue model is improved to consider the effect of electromagnetic induction and radiation by introducing the magnetic flux variable into the model. Memristor is used realize feedback coupling between magnetic flux and membrane potential. The sampled time series and spatial distribution for membrane potentials of myocardial cells in cardiac tissue are investigated by using nonlinear analysis and pattern selection. It is found that the collective behaviors of myocardial cells and electrical modes are much dependent on the distribution of magnetic flux. It indicates that electromagnetic induction can change the formation of spatial patterns and electrical activities of myocardial cells. As a result, the cardiac tissue can be deformed and the wave propagation can be destroyed when it is exposed to electromagnetic radiation. Our present model could be further used to suppress the damage in cardiac tissue induced by electromagnetic radiation. Furthermore, it could also be helpful to detect the collapse of neuronal network, occurrence of disease in brain exposed to electromagnetic field by discussing similar problems in neuronal network^46–50^.
